# Surface Electrical Potentials of Root Cell Plasma Membranes: Implications for Ion Interactions, Rhizotoxicity, and Uptake

**DOI:** 10.3390/ijms151222661

**Published:** 2014-12-08

**Authors:** Yi-Min Wang, Thomas B. Kinraide, Peng Wang, Xiu-Zhen Hao, Dong-Mei Zhou

**Affiliations:** 1 Key Laboratory of Soil Environment & Pollution Remediation, Institute of Soil Science, Chinese Academy of Sciences, Nanjing 210008, China; E-Mails: wangym@issas.ac.cn (Y.-M.W.); xzhao@issas.ac.cn (X.-Z.H.); 2 University of Chinese Academy of Sciences, Beijing 100049, China; 3 Agricultural Research Service, United States Department of Agriculture, Collaborating Scientist, Plant Science Research Unit, Raleigh, NC 27695, USA; E-Mail: tom@kinraide.net; 4 The University of Queensland, School of Agriculture and Food Sciences, St. Lucia, Queensland 4072, Australia; E-Mail: p.wang3@uq.edu.au

**Keywords:** plasma membrane, surface electrical potential, heavy metal, rhizotoxicity, metal uptake, risk assessment

## Abstract

Many crop plants are exposed to heavy metals and other metals that may intoxicate the crop plants themselves or consumers of the plants. The rhizotoxicity of heavy metals is influenced strongly by the root cell plasma membrane (PM) surface’s electrical potential (ψ_0_). The usually negative ψ_0_ is created by negatively charged constituents of the PM. Cations in the rooting medium are attracted to the PM surface and anions are repelled. Addition of ameliorating cations (e.g., Ca^2+^ and Mg^2+^) to the rooting medium reduces the effectiveness of cationic toxicants (e.g., Cu^2+^ and Pb^2+^) and increases the effectiveness of anionic toxicants (e.g., SeO_4_^2−^ and H_2_AsO_4_^−^). Root growth responses to ions are better correlated with ion activities at PM surfaces ({*I^Z^*}_0_) than with activities in the bulk-phase medium ({*I^Z^*}_b_) (*I^Z^* denotes an ion with charge *Z*). Therefore, electrostatic effects play a role in heavy metal toxicity that may exceed the role of site-specific competition between toxicants and ameliorants. Furthermore, ψ_0_ controls the transport of ions across the PM by influencing both {*I^Z^*}_0_ and the electrical potential difference across the PM from the outer surface to the inner surface (*E*_m,surf_). *E*_m,surf_ is a component of the driving force for ion fluxes across the PM and controls ion-channel voltage gating. Incorporation of {*I^Z^*}_0_ and *E*_m,surf_ into quantitative models for root metal toxicity and uptake improves risk assessments of toxic metals in the environment. These risk assessments will improve further with future research on the application of electrostatic theory to heavy metal phytotoxicity in natural soils and aquatic environments.

## 1. Introduction

Contamination of the environment by heavy metals and other intoxicating metals has been studied for many years because of the ubiquity and bioavailability of such metals [1]. Although metals such as Cu, Zn, Mn, Mo, Ni, and Co are essential for plants and animals, some of these metals are well known to be toxic to organisms when present in excessive concentrations [2]. For many years environmentalists have considered the chemical, physiological, and toxicological characteristics of heavy metals in risk assessments and in the formulation of regulatory criteria [3]. However these procedures still focus on the total or free-metal ion activities, as described by the free-metal ion activity model (FIAM), and sometimes ignore some important environmental factors such as pH and the concentrations of ameliorative ions such as Ca^2+^ and Mg^2+^. Di Toro *et al.* have emphasized that toxic cations (commonly heavy metals) compete with ameliorative ions for active sites at the cell plasma membrane (PM) surface [4]. This view of site-specific competition has been incorporated into the biotic ligand model (BLM), and the BLM is commonly accepted by scientists and regulators. The BLM has been applied to several different metals and organisms, and the model is still undergoing improvement.

Although the success of the BLM in risk assessments of cationic toxicants has been demonstrated extensively, its inability to evaluate aspects of anionic toxicity has been noted. Specifically, cationic ameliorants such as Ca^2+^ and Mg^2+^ alleviate the toxicity of cationic toxicants such as Cu^2+^ and Hg^2+^, but these cationic ameliorants enhance the toxicity of anionic toxicants such as SeO_4_^2−^ and H_2_AsO_4_^−^. The latter cannot be interpreted in terms of site-specific competition (*i.e.*, by the BLM). Surely, competition plays a role in the interactions between toxicants and ameliorants, but its role may be small compared to the role played by global (rather than site-specific) electrical potentials at the outer surface of the PM. These variable, but usually negative potentials arise from negatively charged structural components of the PM such as the carboxylic acid groups of acidic amino acids and the phosphate groups of phospholipids [5,6]. These negative charges are the source of the PM surface electrical potential (denoted ψ_0_). ψ_0_ affects the distribution of ions between the bulk-phase solution and the PM surface, thereby playing an important role in plant-ion interactions [7]. The addition of common cations, such as Ca^2+^, Mg^2+^, and H^+^, to the rooting medium reduces ψ_0_ negativity through ion binding and charge screening at the PM [8]. A World-Wide-Web-accessible (Web-accessible) Gouy-Chapman-Stern (GCS) model is now available for the computation of ψ_0_ and ion activities at PM surfaces [9]. A growing number of studies have employed electrostatic theory and the Gouy-Chapman-Stern model to evaluate plant-ion interactions [9,10,11,12].

**Figure 1:**
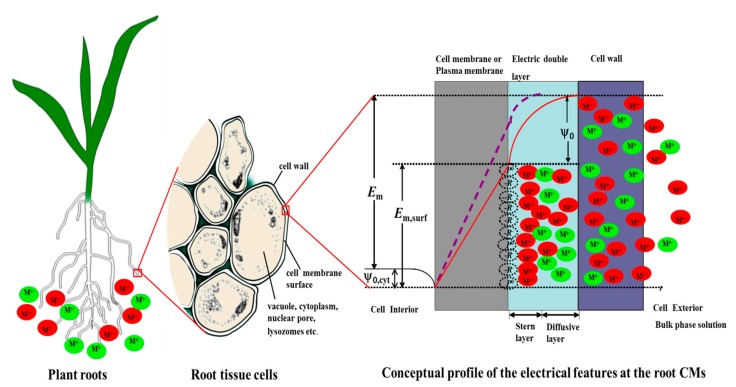
Schematic diagram of electrical profiles and ion distributions at a plant root cell surface, as described in the text. Cations are denoted by M*^z^*^+^ (in red circle), and anions are denoted by M*^z^*^−^ (in green circle). −*R*^−^ and −*P*^0^ denote negatively charged and uncharged membrane components exposed at the outer surface of the plasma membrane (PM). The solid curve represents the profile of electrical potential from the external medium to the inner surface of the PM; The dashed curve illustrates this profile after the addition of surface-depolarizing solutes to the external medium. *E*_m_ is the transmembrane electrical potential difference from the bulk solution to the cell interior; ψ_0_ is the potential difference between the PM outer surface and the external medium; *E*_m,surf_ is the potential difference from the plasma membrane exterior surface to the membrane interior; and ψ_0,cyt_ is the potential difference between the PM inner surface and the cell cytoplasm. ψ_0_ and *E*_m,surf_ are altered, but ψ_0,cyt_ and *E*_m_ remain constant.

These studies demonstrate the advantage of using PM-surface ion activities ({*I^Z^*}_0_, where *I^Z^* denotes an ion with charge *Z*) in the assessment of plant-ion interactions. Responses such as intoxication, alleviation of intoxication, and ion uptake often correlate well with {*I^Z^*}_0_ and often correlate poorly with {*I^Z^*}_b_ (ion activities in the bulk-phase rooting medium) (see [13] for extensive tabulations). Kinraide analyzed the mechanisms of Ca^2+^ alleviation of several ion toxicities, including salinity toxicity, in terms of electrostatic effects. Among these was the Ca^2+^-induced reduction in the negativity of ψ_0_ and the consequent reduced attraction of cationic toxicants to the PM surface [14]. Kopittke *et al.* confirmed the advantage of using {*I^Z^*}_0_ over {*I^Z^*}_b_ in the assessment of cowpea root responses to Cu or Pb [11]. Electrostatic effects also proved to be essential for modeling the rhizotoxicity of SeO_4_^2^^−^ and H_2_AsO_4_^−^ [15,16]. As we shall illustrate later, changes in ψ_0_ influence not only the value of {*I^Z^*}_0_, they also influence the gradient of electrical potential across the PM from outer surface to inner surface (denoted *E*_m,surf_). The largest component of commonly negative *E*_m,surf_ is *E*_m_, which is the potential difference between the external cell bathing medium and the cell interior (Figure 1). *E*_m,surf_ is responsible for ion-channel voltage gating and is the principal driving force for ion fluxes across the PM [13,17,18]. Thus, in our review study, we aimed to (1) introduce the basic theory of cell-surface electrostatic effects; (2) specify the role of ψ_0_ in plant responses to heavy metal stress; and (3) consider the prospect of additional use of electrostatic theory in future assessments of organism-ion interactions in natural soil environments.

## 2. Basic Theory of Gouy-Chapman-Stern (GCS) Model

Two search engines (ISI Web of Science and Google Scholar) were used for the acquisition of data from the period 1964–2014. These data were concerned mainly with solution-culture studies of the toxicity and uptake of Zn, Cd, Pb, Cu, Co, Ni, Al, SeO_4_^2−^, and H_2_AsO_4_^−^. These studies considered electrostatic effects and the influence of different environmental factors, such as pH and the concentrations of Ca, Mg, Na, and K, in modeling responses to toxicants. A variety of species were used in the studies; they included wheat, barley, pea, cowpea, lettuce, *Escherichia coli*, and phytoplankton.

### 2.1. Computation of the Cell-Surface Potential (ψ^0^) with a GCS Model

The Gouy-Chapman-Stern model is composed of Gouy-Chapman theory (a century-old electrostatic theory) and the Stern portion of the model, which incorporates ion binding to the PM surface. This GCS model enables the computation of ψ_0_ and, from that, the ion activities at the PM surface ({*I^Z^*}_0_). Details of this GCS model can be found in a book chapter [19] and several published papers [7,20,21]. A brief review of the model is presented here.

In the Gouy-Chapman theory, the intrinsic surface charge density (σ_0_, in units Coulombs per m^2^) at the PM surface is the total charge density in the absence of ion binding. If the PM surface binding sites (*i.e.*, negatively charged sites *R*^−^ and neutral sites *P*^0^) are occupied by ions, then the actual PM-surface charge density (*σ*) may be expressed by the Grahame Equation:

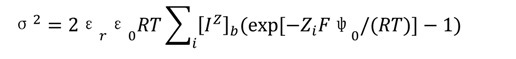
(1)
where *2 ε _r_ ε _0_RT* = 0.00345 when concentrations are expressed in M at 25 °C (ε_r_ is the dielectric constant for water, ε_0_ is the permittivity of a vacuum, and *F*, *R*, and *T* are the Faraday constant, the gas constant, and the temperature, respectively ). [*I*^Z^]_b_ refers to free ion concentrations in the bulk-phase medium, and *Z*_i_ is the charge on the i*th* ion.

Ion binding (the Stern modification) produces the PM-surface species *RI^Z^*^−1^ and *PI^Z^*, and equilibrium reactions may be written as follows:

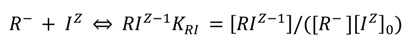
(2)

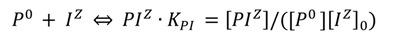
(3)
where [*R*^−^], [*P*^0^], [*RI*^Z−1^] and [*PI*^Z^] denote PM surface densities expressed in mol·m^−2^. [*I*^Z^]_0_ denotes the concentration of the unbound ion *I^Z^* at the PM surface. Therefore, taking the Stern modification of the Gouy-Chapman theory into consideration, the actual σ in Equation (1) can also be expressed as:

σ = {−[*R*^−^] + ∑*_i_*(*Z_i_* − 1)[*RI^Z^*^−1^] + ∑*_i_Z_i_*[*PI^Z^*]}*F* (4)

To calculate the values of [*I^Z^*]_0_, a Boltzmann Equation is introduced:

[*I^Z^*]_0_ = [*I^Z^*]_b_ exp[−*Z_i_F*ψ_0_/(*RT*)] (5)

To compute ψ_0_, trial values (changed incrementally and progressively) are assigned to ψ_0_ in Equations (1) and (5) until the values for σ computed in Equations (1) and (4) converge. Of course, one must have values for other parameters such as the equilibrium constants in Equations (2) and (3) and the total surface densities of binding sites *R* and *P*. These values are available in the Web-accessible GCS model [9]. After that, knowledge of ψ_0_ enables the calculation of ion activities at the PM surface ({*I^Z^*}_0_) according to the Nernst Equation:

{*I^Z^*}_0_ = {*I^Z^*}_b_ exp[−*Z_i_F*ψ_0_/(*RT*)] (6)

where {*I^Z^*}_b_ is the free ion activity of the i*th* ion in the bulk-phase medium. These values may be obtained from dedicated speciation programs (e.g., Visual Minteq, WHAM, GEOCHEM, or PhreeqcI) and from the Web-accessible GCS model.

### 2.2. Data Analysis

Under heavy metal stress, responses of organisms were recorded and analyzed for toxicity assessment. For plant growth, root growth inhibition was usually used for metal rhizotoxicity assessment. For bacteria, potential nitrification rate and glucose-induced respiration were evaluated [22]. These responses were then incorporated into a modified Weibull Equation. In the equation below, relative root elongation (RRE) in the presence of toxicant is expressed as a percent of root elongation (RE) in the absence of toxicant (*RRE* = 100*RE*_toxicant present_/*RE*_toxicant absent_):

*RRE* = 100/exp[(*a*{*T*}_0_)*^b^*] (7)

where {*T*}_0_ is the toxicant intensity (activity in our review) at PM surface. Coefficient *a* is the strength coefficient and increases with the strength of the toxicant, and *b* is a shape coefficient. When *b* > 1, the plot of *RRE vs.* {*T*}_0_ is downwardly sigmoidal. When {*T*}_0_ is very great, *RRE* = 0%; when {*T*}_0_ is zero, *RRE* = 100%. Coefficients *a* and *b* are determined by regression analysis of paired values of *RRE* and {*T*}_0_.

## 3. Application of Cell-Surface Electrical Potential in Studies of Metal Toxicity

### 3.1. The Profile of Electrical Potential across the Cell Surface

For plant PMs, three global electrical properties account for the plant-metal interactions. Figure 1 illustrates a cell in which the PM and the cell wall (CW) are separated because of mild plasmolysis; that is, the plant tissue is slightly wilted. The gap between the PM and the CW is considered to be filled with an aqueous solution similar to, but not identical to, the external bathing medium. In soil-grown plants, the external bathing medium and the gap solution are grounded naturally, and in experimental work these solutions are usually, but not necessarily, grounded deliberately. The transmembrane electrical potential difference (*E*_m_) is the electrical potential difference from the bulk solution to the cell interior. *E*_m_ is the sum of three potential differences. One of these is the potential difference between the PM outer surface and the external medium (ψ_0_). The notation ψ_0_ indicates the potential at zero distance from the PM surface. The curved lines represent the profile of the electrical potential upon approach to the PM surface. The second potential difference is denoted *E*_m,surf_. This is the potential difference from the PM exterior surface to the PM interior surface. As a simplification, the profile of *E*_m,surf_ across the PM is depicted as a straight line. The third potential difference (ψ_0,cyt_) is the potential difference between the PM inner surface and the cell cytoplasm. ψ_0_ is responsive to the ionic composition of the bulk medium; ψ_0,cyt_ ≈ 10 mV and is unresponsive to the ionic composition of the bulk medium. Consequently, *E*_m,surf_ is linearly related to ψ_0_. Because *E*_m,surf_ controls ion-channel voltage gating and provides the driving force for ion influx across the PM surface, both gating and driving force are effectively controlled by ψ_0_.

The preceding discussion may be appropriate for mildly plasmolyzed cells or for cell protoplasts from which the CW has been removed enzymatically. However, in the case of turgid, intact cells, the PM is pressed tightly against the CW. In such cases, it may not be reasonable to assume that the cell may be modeled as though the CWs have no influence upon ψ_0_ and {*I^Z^*}_0_. In fact, the structural components of the CWs carry negative charges, and the aqueous solution within the CWs is commonly considered to be in Donnan equilibrium with the external bathing media. Consequently the CW Donnan phase is negatively charged and enriched in free cations and depleted in free anions relative to the external medium. Shomer *et al.*, [23] have computed and measured indirectly the potential of the CW Donnan phase relative to the external medium (ψ_CW_) and Kinraide [24] has analyzed the possible effect of the CWs upon ψ_0_ and {*I^Z^*}_0_ after assuming that the PM surface is bathed in the CW Donnan phase (Model 2) rather than in the external medium (Model 1). Values for ψ_0,Model 1_ were surprisingly similar to the values computed for ψ_0,Model 2_. For 16 solutions orthogonal for the solutes CaCl_2_ (0.1 or 1.0 mM), NaCl (1 or 10 mM), LaCl_3_ (1 or 10 μM), and H^+^ (pH 4.6 or 5.6). *r*^2^ = 0.989 for the regression ψ_0,Model 1_ = *a* + *b* ψ_0,Model 2_, where coefficient *a* = 0.001 and coefficient *b* = 1.000 are both significant. To compute ion activities at PM outer surfaces according to Model 1 or Model 2, one incorporates either ψ_0,Model 1_ and {*I^Z^*}_b_ or ψ_0,Model 2_ and {*I^Z^*}_CW Donnan phase_ into the Nernst Equation (Equation (6)). The resulting values for {*I^Z^*}_0,Model 1_ and {*I^Z^*}_0,Model 2_ are as highly correlated as the values for ψ_0,Model 1_ and ψ_0,Model 2_. The explanation for these similarities in values is that the placement of CWs against PMs causes multiple offsetting effects, as described in detail in Kinraide [24]. On the basis of these analyses and on the basis of experimental work cited in the study of Kinraide [24], one may compute the electrostatic effects upon toxicity, the alleviation of toxicity, and the uptake of ions on the basis of ψ_0_ and {*I^Z^*}_0_ computed as though CWs had no effect (Model 1). These criteria can be applied to evaluate heavy metal rhizotoxicity and accumulation in metal-polluted soils.

### 3.2. Verification of the Calculated Values of ψ^0^, and Some Parameter Values of the GCS Model

ψ_0_ is difficult to measure, whereas the measurement of *E*_m_ is relatively simple. For the latter, measurement entails the insertion of microelectrodes into cells [7,25]. Values for ψ_0_ are commonly computed from ζ potentials. These are the near-surface electrical potentials measured in electrophoresis experiments. ζ potentials are well correlated with calculated ψ_0_ values (see Table S1 and see [26]). The calculated ψ_0_ and the measured ζ potential in different media from previous studies are listed in Table S1. Physiological responses to ionic solutions provide indirect evidence for the accuracy of computed values for ψ_0_.

As described by the GCS model above, computation of ψ_0_ requires the knowledge of σ_0_ (units: Coulombs per m^2^) and the ionic composition of the rooting medium. Kinraide and Wang [26] considered σ_0_ to be the parameter in greatest doubt, and in a dedicated analysis of published data obtained by different experimental and computational methods concluded that σ_0_ = −30 mC·m^−2^ is a suitable value for PMs generally, despite small differences obtained from different species and different cultural conditions. Computation of ψ_0_ also requires estimated values for ion binding strength to the negative and neutral binding sites (*R*^−^ and *P*^0^) at the PM surface. Yermiyahu and co-workers [19,21] used ion adsorption experiments to compute binding strengths for 49 ions. Later, Kinraide and Yermiyahu [27] presented “a scale of metal ion binding strengths correlating with ionic charge, Pauling electronegativity, toxicity, and other physiological effects”. Eventually, an algorithm to compute the binding strengths of 64 ions was developed [28]. Some of these ions accumulate excessively in agricultural soils.

### 3.3. Cationic Toxicants

**Figure 2:**
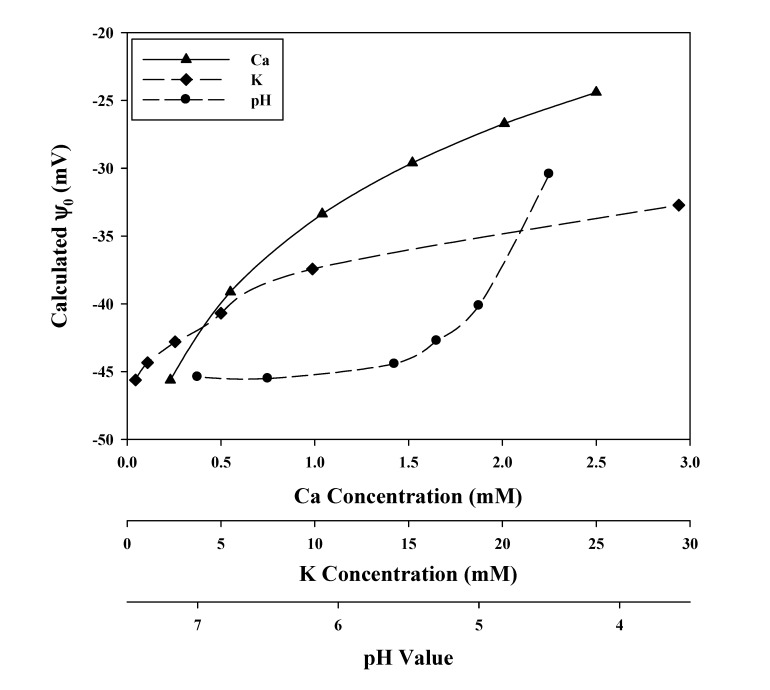
Responses of root cell plasma membrane (PM) surface electrical potential (ψ_0_) to increasing concentrations of Ca^2+^, K^+^, and H^+^ in the cell-bathing media, which contain a background of 0.23 mM Ca^2+^, 0.22 mM Mg^2+^, 0.97 mM Na^+^, 0.44 mM K^+^ and pH = 6.0. These ψ_0_ values were calculated by the Gouy-Chapman-Stern (GCS) model. Data were obtained from Wang *et al.* [29].

**Figure 3:**
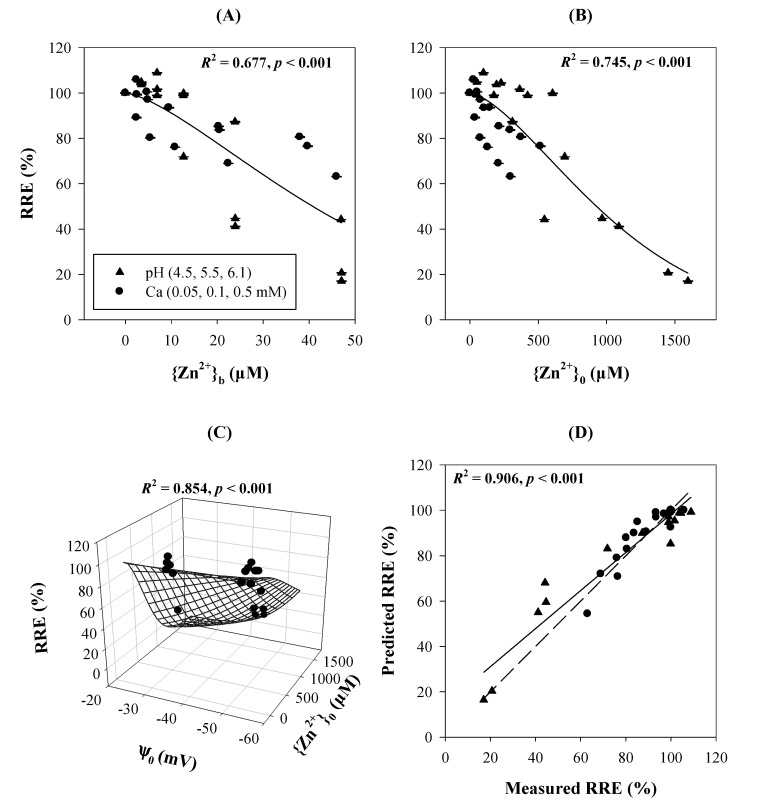
Relative root elongation (RRE, %) for wheat seedlings in response to Zn^2+^ treatments under different pH values (4.5, 5.5, and 6.1) or Ca^2+^ concentrations (0.05, 0.1, and 0.5 mM). Metal ion activities were expressed as ion activities in bulk-phase solutions ({Zn^2+^}_b_) or activities at the PM surface ({Zn^2+^}_0_). The equation *RRE* = 100/exp[(*a*{Zn^2+^}_b_)*^b^*] (Equation (8)) was used in (**A**); *RRE* = 100/exp[(*a*{Zn^2+^}_0_)*^b^*] (Equation (9)) was used in (**B**); *RRE* = 100/exp[(*a*(1 + *c* ψ_0_){Zn^2+^}_0_)*^b^*] (Equation (10)) was used in (**C**); and *RRE* = 100{1 − 1/exp(*p*{Ca^2+^}_0_)}/exp[(*a*(1 + *c* ψ_0_){Zn^2+^}_0_)*^b^*] (Equation (11)) was used in (**D**). The dashed line in (**D**) shows the 1:1 slope relationship. Data were obtained from Wang *et al.* [12,35].

Metallic toxicants carrying positive charges are concentrated at the PM surface due to the negative ψ_0_. For instance, when ψ_0_ = −50.0 mV, mono-, di-, and trivalent cations will be concentrated at 7-, 49-, and 343-fold at the PM surface, respectively. These attracted cations reduce the negativity of ψ_0_ through binding and electric screening at the PM surface. The GCS model can compute these changes, and the calculated ψ_0_ values in response to added cations are shown in Figure 2. For example, increasing bulk-phase Ca^2+^ from 0.23 to 2.50 mM increased (reduced the negativity of) calculated ψ_0_ from −45.6 to −24.4 mV; increasing K^+^ from 0.44 to 29.4 mM increased ψ_0_ from −45.6 to −32.7 mV; and reducing pH from 7.0 to 4.5 increased ψ_0_ from −45.4 to −30.4 mV. The reduced negativity of ψ_0_ values would reduce the attraction of cationic toxicants at the PM surface, resulting in the alleviation of the cationic metal toxicity. The magnitude of the ψ_0_ increase was strongly dependent on the ion charge and the strength of binding to the PM surface. In a typical growth experiment with variable concentrations of Zn and other cations, shown in Figure 3, we demonstrated the effectiveness of ψ_0_ in the modeling of rhizotoxicity. When relative root elongation (RRE) was expressed as a function of {Zn^2+^}_0_ (Equations (8) and (9)), a better correlation was found (*r*^2^ = 0.745, *p* < 0.0001, *n* = 36 (Figure 3B)) than when *RRE* was expressed as a function of {Zn^2+^}_b_ (*r*^2^ = 0. 677, *p* < 0.0001, *n* = 36 (Figure 3A)). Incorporation of both {Zn^2+^}_0_ and ψ_0_ into the Weibull Equation (Equation (7) elevated *r*^2^ to 0.854 (*p* < 0.0001, *n* = 36 (Equation (10), Figure 3C)). Moreover, incorporation of {Ca^2+^}_0_ into the model (*p* < 0.0001, *n* = 36 (Equation (11), Figure 3D)) further increased the *r*^2^ to 0.906. Ca is well known as an essential nutrient in agricultural fields and as an ameliorant. Kinraide [14] proposed three mechanisms by which Ca^2+^ alleviates the toxicity of Al^3+^, H^+^, and Na^+^. These mechanisms included (1) reduced cationic toxicant activities at PM surface due to the decreased ψ_0_ negativity by Ca addition; (2) the restoration of Ca^2+^ activity at PM surface if surface Ca has been reduced to growth-limiting levels by the toxicant; and (3) an assortment of other mechanisms such as the possible competition between Ca and toxicants at the PM surface or the blocking of the PM channels by Ca addition [17]. Some polyvalent, strongly binding ions, such as Al^3+^ and La^3+^, have a direct effect on ψ_0_ change even if they appear to be channel blockers [17,21]. More studies further demonstrated that intoxication (e.g., reduced root elongation, reduced bacterial nitrification rate, and reduced glucose-induced respiration) can be better explained by the metallic ion activities at the PM surface rather than by ion activities in bulk-phase medium and other electrostatic effects [11,22].

Metal species other than the naked (unhydrolyzed, uncomplexed) free metal ions may be toxic. Wagatsuma and Ezoe [30] showed that the pH of the medium controls both the identity of the Al species and the toxicity of Al and argued that hydroxo-Al polymer ions are more toxic to plant roots than mononuclear Al ions. Studies by Parker *et al.* [31] confirmed that the polynuclear hydroxo-Al (Al_13_) complex was at least 10-fold more rhizotoxic than Al^3+^ to wheat seedlings. Possibly due to ion binding strengths at the PM surface, the rhizotoxicity of Al species to wheat roots follows the order: Al_13_ > Al^3+^ > AlF^2+^ > AlF_2_^+^ [32]. Wang *et al.* [33], determined that when the pH values of the nutrient solutions ranged from 4.50 to 8.25, Cu^2+^ and CuCO_3_ both contributed to the inhibition of wheat root growth, which was well modeled by taking these species’ activities at the PM surface into consideration.

**Figure 4:**
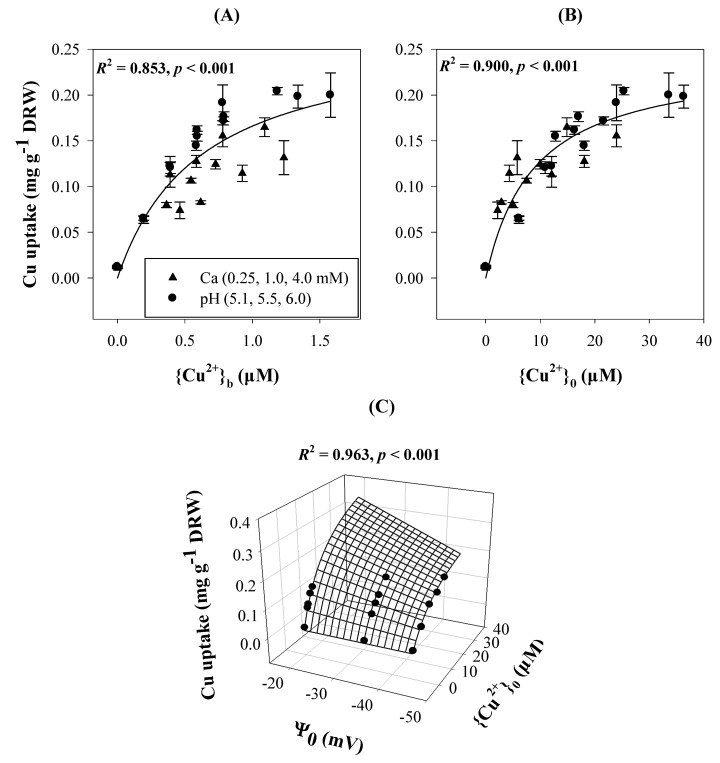
Metal accumulation in wheat seedling roots in response to Cu^2+^ treatments under different values for pH (5.1, 5.5, and 6.0) and Ca concentration (0.25, 1.0, and 4.0 mM). Metal ion activities were expressed as ion activities in bulk-phase solutions ({Cu^2+^}_b_) or activities at the PM surface ({Cu^2+^}_0_). The Michaelis-Menten Equation Cu uptake = *a*{Cu^2+^}_b_/(*K*_m_ + {Cu^2+^}_b_) (Equation (12)) was used in (**A**); Cu uptake = *a*{Cu^2+^}_0_/(*K*_m_ + {Cu^2+^}_0_) (Equation (13)) was used in (**B**); and Cu uptake = *a*(1 + *b* ψ_0_){Cu^2+^}_0_/(*K*_m_ + {Cu^2+^}_0_) (Equation (14)) was used in (**C**). Data were obtained from Wang *et al.* [13].

Intracellular metal uptake entails ion diffusion to and accumulation at the PM surface followed by influx of the ion across the PM, sometimes through transporters such as those for Cu [34]. Both surface accumulation and influx are influenced by ψ_0_, the latter because *E*_m,surf_ is a linear function of ψ_0_. As illustrated in Figure 4, using {Cu^2+^}_0_ in the traditional Michaelis-Menten Equation for metal uptake modeling resulted in a higher correlation (*r*^2^ = 0.900, *p* < 0.0001, *n* = 30 (Equation (13), Figure 4B)) than using {Cu^2+^}_b_ (*r*^2^ = 0.853, *p* < 0.0001, *n* = 30 (Equation (12), Figure 4A)). Moreover, incorporation of both {Cu^2+^}_0_ and ψ_0_ into the Michaelis-Menten Equation demonstrates the dual roles of ψ_0_ on metal internalization, and significantly increased *r*^2^ from 0.853 to 0.963 (*p* < 0.0001, *n* = 30 (Equation (14), Figure 4C)). Detailed descriptions of the dual effects of ψ_0_ on metal ion uptake and toxicity are presented in Kinraide [17] and Wang *et al.* [13]. Thus, an electrostatic uptake model was developed to incorporate both ψ_0_ and surface ion activities for ion uptake [13].

### 3.4. Anionic Toxicants

In contrast to the extensive studies of cationic toxicants, anionic toxicants have been little studied in terms of toxicity modelling. As mentioned in the Introduction, the BLM model fails to assess anionic metalloid bioavailability. Due to the dominance of anionic forms in many metalloids, Kinraide and co-workers [15,16] introduced ψ_0_ into the interpretation and modeling of anionic toxicants, in particular SeO_4_^−^, and H_2_AsO_4_^−^.

**Figure 5:**
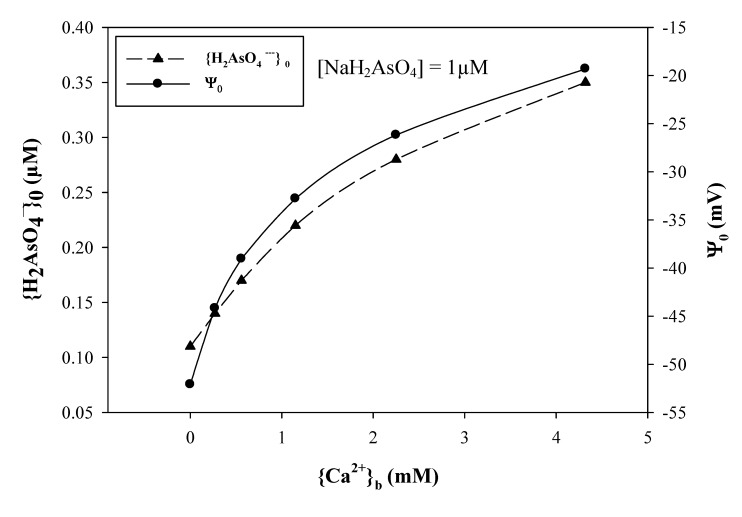
The calculated PM surface activities of H_2_AsO_4_^−^ ({H_2_AsO_4_^−^}_0_) at 1.0 µM NaH_2_AsO_4_, and the calculated PM surface electrical potential (ψ_0_) in response to different Ca^2+^ activities ({Ca^2+^}_b_) in test solutions, which contain a background of 0.27 mM Ca^2+^, 0.26 mM Mg^2+^, 1.26 mM Na^+^, and 0.52 mM K^+^ at pH 6.0.

**Figure 6:**
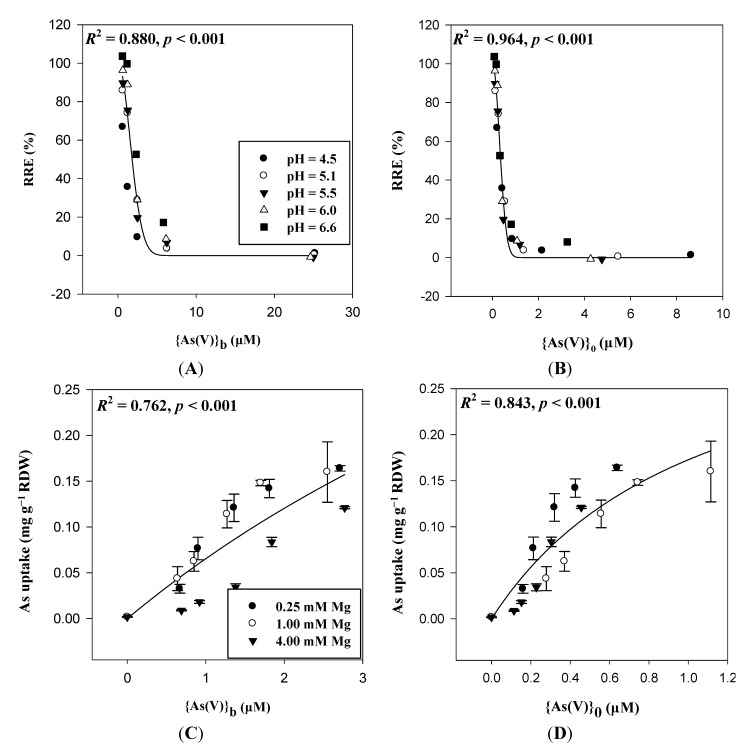
Relative root elongation (RRE, %) and root metal accumulation in wheat seedlings in response to NaH_2_AsO_4_ treatments under different pH and Mg levels. Ion activities were expressed as activities in the bulk-phase solutions ({As(V)}_b_) and at the PM surface ({As(V)}_0_). The Weibull Equation RRE = 100/exp[(*a*{As(V)}_b_)*^b^*] (Equation (15)) was used in (**A**); and the Equation RRE = 100/exp[(*a*{As(V)}_0_)*^b^*] (Equation (16)) was used in (**B**); The Michaelis-Menten equation as Uptake = *a*{As(V)}_b_/(*K*_m_ + {As(V)}_b_) (Equation (17)) was used in (**C**); and the equation as Uptake = *a*{As(V)}_0_/(*K*_m_ + {As(V)}_0_) (Equation (18)) was used in (**D**). Data were obtained from Wang *et al.* [13,29].

In Figure 5, we see that when {Ca^2+^}_b_ was increased from 0 to 4.32 mM, ψ_0_ increased from −52.1 to −19.3 mV. The reduced negativity of ψ_0_ concentrated {H_2_AsO_4_^−^}_0_ from 0.11 to 0.35 µM when the test solutions contained 1 µM NaH_2_AsO_4_. This resulted in the enhancement of the As(V) rhizotoxicity. In Figure 6 we present wheat seedling root growth and root metal uptake in response to H_2_AsO_4_^−^. Under different pH levels in the test solutions, *RRE* was better correlated with {As(V)}_0_ (*r*^2^ = 0.964, *p* < 0.0001, *n* = 25 (Figure 6B, Equation (16))) than with {As(V)}_b_ (*r*^2^ = 0.880, *p* < 0.0001, *n* = 25 (Figure 6A, Equation (15))). Traditional ion competition for binding sites could not provide a reasonable explanation of toxicity, but the PM surface activities of As(V) ({As(V)}_0_) well interpreted the results of As(V) toxicity. In modeling the As(V) root uptake, we also found that substitution of {As(V)}_0_ for {As(V)}_b_ improved the prediction of uptake from an *r*^2^ of 0.762 (*p* < 0.0001, *n* = 18 (Figure 6C, Equation (17))) to an *r*^2^ of 0.843 (Figure 6D, *p* < 0.0001, *n* = 18 (Equation (18))). Related results have been reported by Kinraide [15], who detected an enhancement of SeO_4_^−^ rhizotoxicity from decreases in pH or from additions of CaCl_2_, MgCl_2_, or SrCl_2_.

Despite the superiority of using ψ_0_ in the evaluation of metalloid rhizotoxicity, we should keep in mind that some metalloid species are uncharged. In some cases, ion-specific interactions at PM, such as channel blockade, more than the global electrostatic effect, may play important roles in toxicity and its alleviation. Therefore, efforts to clarify additional mechanisms and to improve models should continue in future studies [36].

## 4. Ongoing Use of Electrostatic Principles in Risk Assessment and Modeling

For decades, ψ_0_ has used extensively to assess the effects of single toxicants in the environment. However, environmental management should be based on the risk assessment of multiple metal toxicants in many cases. In natural environments, organism exposure to metal mixtures has been reported broadly. For example, An *et al.*, reported that when cucumber (*Cucumis sativus*) was exposed to Cu-Cd mixtures, Cu accumulation in shoots was inhibited by Cd [37]. Cui *et al.*, investigated human urine and blood as indicators of health after exposure to Cd-Pb mixtures in Nanning (China). The results showed that the mixtures caused significant renal dysfunction in the residents [38]. Moreover, studies of multiple metal toxicity in soil invertebrates (earthworms, nematodes, *etc.*) and microorganisms (*Escherichia coli*, *Pseudomonas fluorescens*, *etc.*) have been reported [39,40].

**Figure 7:**
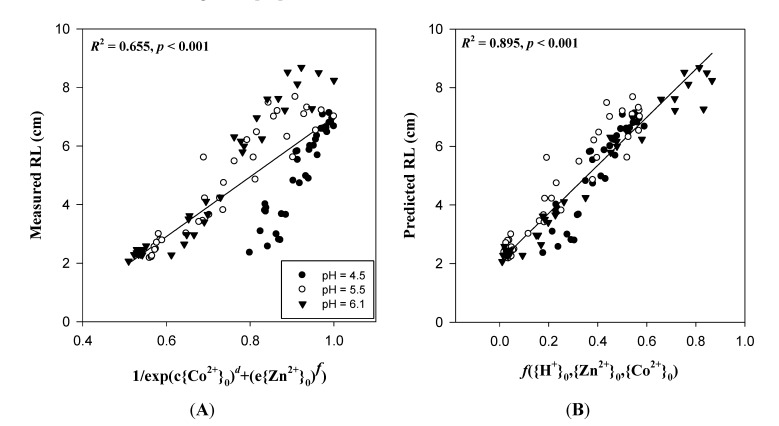
Comparison of the measured (**A**) and predicted (**B**) root length (RL) when wheat seedlings were exposed to Zn-Co mixtures. Predicted RL was based upon extended multiplicative models using ion activities at PM surfaces ({Zn^2+^}_0_ and {Co^2+^}_0_). The solid lines show the linear regression. The details of the coefficients *c* and *e* (Equation (19)) and the function *f*({H^+^}_0_,{Zn^2+^}_0_,{Co^2+^}_0_) (Equation (20)) are described in Table S2. Data were obtained from Wang *et al.* [12].

Currently, joint action assessments of multiple metals are commonly based on the traditional TU (toxic unit) concept combined with the CA (concentration addition) or IA (independent action) models. Vijver *et al.*, showed that metal combinations, antagonistic and synergistic effects are usually observed in toxic-metal analyses [41,42]. Therefore, the essential scientific challenge is to further develop the traditional models for responses to multiple metals. Le *et al.*, [43] introduced the traditional CA concept into the BLM model and successfully evaluated the effect of binary mixtures of Cu-Ag and Cu-Zn toxicity on lettuce, *Lactuca sativa*. However, these studies of metal mixtures are still based on free metal ion activities or concentrations in the external media rather than metal activities at PM surfaces. In Figure 7, we present wheat seedling root growth in response to Zn-Co mixtures. Considering ion interactions among Zn^2+^, Co^2+^, and H^+^ at the PM surface enhanced the prediction ability (*r*^2^) from 0.655 (*p* < 0.0001, *n* = 108 (Figure 7, Equation (19) in Table S2)) to 0.895 (*p* < 0.0001, *n* = 108 (Figure 7, Equation (20) in Table S2)), based on an extended multiplicative model. Details of the correlations are shown in Table S2. Kinraide [44], in a study of salinity toxicity, used a multiplicative model to quantitatively assess the multiple toxic and ameliorative effects of PM surface activities of Ca^2+^, Na^+^ and K^+^. Hence, we should recognize the potency of ψ_0_ for the assessment of multi-metal toxicity in the environment [12,35].

Furthermore, environmental risk evaluation is a difficult task in the natural soil environment due to the complexity of soil properties. Previous studies have principally used plants grown in solution culture. Developed methods for collecting soil solutions and calculating metal speciation in soil solutions (e.g., programs such as WHAM-humic software and the GCS program) have enabled the application of electrostatic principles in soil environmental risk assessment. Wang *et al.* [45] applied an electrostatic toxicity model (ETM) for modeling Ni^2+^ toxicity to barley root growth in soils. The results indicated that root elongation was well correlated with the {Ni^2+^}_0_, {Ca^2+^}_0_, {Mg^2+^}_0_, and the osmotic effects of soil solutions. Thus, the developed ETM model has the potential to assess the risk of metal toxicity in terrestrial ecosystems. Soil microorganisms (e.g., *Escherichia coli* and rhizobia) and soil invertebrates (e.g., earthworms) also face heavy metal stresses. Wang *et al.*, [22] established parameters in the GCS model for *Escherichia coli*, and showed that both ψ_0_ and metal ion activities ({Cu^2+^}_0_ and{Ni^2+^}_0_) at the outer surfaces of bacterial cell membranes influenced the variation in potential nitrification rate (PNR) and the glucose-induced respiration (GIR). Therefore, we conclude that electrostatic effects must be taken into account in the evaluation of metal environmental risk in soil ecosystems and that ongoing research should continue to improve the electrostatic approach.

## Supplementary Materials

Supplementary tables can be found at http://www.mdpi.com/1422-0067/15/12/22661/s1.
